# Coalitional Distributed Model Predictive Control Strategy for Vehicle Platooning Applications

**DOI:** 10.3390/s22030997

**Published:** 2022-01-27

**Authors:** Anca Maxim, Constantin-Florin Caruntu

**Affiliations:** Department of Automatic Control and Applied Informatics, “Gheorghe Asachi” Technical University of Iasi, 700050 Iasi, Romania; anca.maxim@ac.tuiasi.ro

**Keywords:** coalitional model predictive control, distributed model predictive control, robust positively invariant sets, closed-loop stability, vehicle platooning

## Abstract

This work aims at developing and testing a novel Coalitional Distributed Model Predictive Control (C-DMPC) strategy suitable for vehicle platooning applications. The stability of the algorithm is ensured via the terminal constraint region formulation, with robust positively invariant sets. To ensure a greater flexibility, in the initialization part of the method, an invariant table set is created containing several invariant sets computed for different constraints values. The algorithm was tested in simulation, using both homogeneous and heterogeneous initial conditions for a platoon with four homogeneous vehicles, using a predecessor-following, uni-directionally communication topology. The simulation results show that the coalitions between vehicles are formed in the beginning of the experiment, when the local feasibility of each vehicle is lost. These findings successfully prove the usefulness of the proposed coalitional DMPC method in a vehicle platooning application, and illustrate the robustness of the algorithm, when tested in different initial conditions.

## 1. Introduction

One of the recent interests in the control community is focused on developing advanced control techniques in the framework of autonomous vehicles. Thus, considerable efforts were made to increase the efficiency and to improve the collaboration through the connectivity between multiple autonomous vehicles, using well established control strategies, such as Model Predictive Control (MPC) or Distributed Model Predictive Control (DMPC) [1]. Among these strategies, vehicle platooning of connected and autonomous vehicles has increased potential [2]. The idea behind this methodology is to control a group of vehicles, such that the entire ensemble travels with a constant velocity, while maintaining a desired, safe inter-vehicle distance [3]. One of the most common vehicle platooning frameworks uses the predecessor-following communication topology. Each platoon vehicle receives information only from its preceding vehicle, via a vehicle-to-vehicle (V2V) communication technology [4].

For vehicle platooning applications, several control approaches were proposed in the state-of-the-art literature, see the review paper [5]. Thus, a distributed adaptive robust $H_{\infty }$ control strategy for vehicle platooning is given in [6]. Here, an adaptive robust $H_{\infty }$ controller is compared in simulation with a robust $H_{\infty }$ controller in a seven vehicles platoon application. The platoon stability is formulated using a Linear Matrix Inequality (LMI) condition derived from the Lyapunov stability theory. Another approach, based on stochastic dynamic programming is provided [7]. The idea is to describe the coordinated platooning problem of autonomous vehicles at highway junctions as a Markov decision process. In [8], a decentralized multi-agent game-theoretical strategy is proposed, in which individual autonomous vehicles form platoons using either one-to-one or many-to-many coordination. Furthermore, two game-theoretical models are proposed, with complete or incomplete information exchanged between agents. The method is validated in simulation, in various tests in which, seven to twenty-five vehicles need to coordinate and form different platoons of maximum size of seven vehicles. In [9], a dynamic event-triggered control and communication strategy is presented. The algorithm is suitable to reduce the utilization of the wireless communication network and ensures a $L_{2}$ string-stable platoon. The methodology that was tested in the simulation is a three vehicle platoon. Furthermore, in [10], a DMPC strategy for vehicle platooning with different types of communication delays is proposed. In [11], a min-max DMPC strategy for vehicle platoons with communication delays is presented. The algorithm provides input-to-state stability margins for the platoon analysis. Each vehicle from the platoon is modelled using a point-mass model, which is the simplest model available, that ignores the vehicle dynamics. In [12], a non-iterative DMPC strategy for flexible vehicle platooning is given. The algorithm implements a vehicle partitioning procedure, suitable when an individual vehicle wants to join an existing platoon. The methodology assumes the platoon partition into different disjoint vehicle clusters, connected through connecting vehicle clusters, which act as common neighbours between the consecutive disjoint clusters. The algorithm is tested on a point-mass vehicle model. In [13], a DMPC algorithm for vehicle platoons with switching communication topologies is presented. Starting from the basic predecessor-leader communication topology, the algorithm switches to another topology, when a communication failure occurs. Thus, if the leader vehicle can not send information, the first follower becomes the new leader of the platoon, whereas when a follower vehicle fails to send information, this is automatically excluded from the platoon communication. The latter will act as an independent vehicle, which physically is still in the platoon, but must rely on its own sensors to maintain a constant inter-vehicle distance with its successor. The string stability analysis of the platoon is formulated in $L_{2}$-norm of the spacing error. The platoon is also modelled with a point-mass vehicle model.

The DMPC method emerged from the mature MPC field and is suitable for complex systems that can be decomposed into smaller interconnected sub-systems. To relieve the computational burden of the methodology, each sub-system is locally controlled by an agent, which solves a smaller optimization problem depending on both local measured information acquired by the sensors and data exchanged within a neighbourhood. This means that all agents corresponding to sub-systems, which are coupled (dynamically or through the constraints or the cost functions), need to exchange relevant information, to ensure local feasibility for the optimization problems [14,15].

Another recent development within this methodology is the Coalitional Control, with characteristic features from cooperative game-theory [16]. The idea is to consider that the interconnected sub-systems have a dynamic communication architecture, which can enable or disable the existing communication links, according to the specific overall control objectives [17].

Following the idea of a flexible communication architecture between coupled sub-systems, a Coalitional Distributed Model Predictive Control (C-DMPC) strategy for a cyber-physical multi-agent application was proposed in [18]. The key feature for this methodology is to use the advantages provided by the DMPC algorithm by creating an adjustable controller framework. The architecture starts from a non-cooperative DMPC structure (i.e., the agents solve local optimization problems taking into account their respective interactions), and evolves into coalitional DMPC, e.g., groups of agents merge in a single entity or cooperative group (hereafter called a coalition) and commonly solve a global problem with respect to the coalition objective [18,19].

Regardless if the DMPC or the C-DMPC framework is considered, since both are derived from the MPC strategy, they inherit one key feature, namely the accommodation of constraints directly during the optimal-design procedure [20,21]. From the dynamical stability point of view, one method to ensure the stability of the MPC algorithm (and implicitly the C-DMPC strategy) is formulating it using the terminal region constraint framework, which can be defined as an invariant set [22,23]. There is a high interest in the literature to develop reliable methods for computing invariant sets for uncertain systems, in which the disturbance acts as an uncertainty in the nominal model [24,25]. Several approaches to calculate robust control invariant sets can be mentioned, such as those based on: graph theory and algorithms [26], linear matrix inequalities [27], Hamilton–Jacobi reachability framework [28] or dynamic programming [29]. In [30], the boundary of the maximal robust positively invariant set is studied by adapting the theory of barriers formulation. The latter is then used in [31], to perform a transient stability analysis for power systems. In [32], an analysis regarding the scalability of robust positively invariant sets with respect to the disturbance set is provided. In [33], robust positive invariant sets are computed using sequence-based techniques and tested on a switched system with average dwell time.

In this work, the focus is on developing and testing a novel C-DMPC methodology. The algorithm is designed for state-coupled sub-systems, in which the interaction between consecutive modules is considered an uncertainty in the states, rather than the input-coupled C-DMPC formulation from [18]. Emphasis is given to the computational procedure used to ensure the closed-loop stability of the algorithm, thus providing the technical details regarding the computation of the maximal robust positively invariant sets, instead of giving the basic pseudo-code algorithm [18]. Hence, to ensure several available options for the terminal region constraint set, a predefined table is computed in the initialization stage of the C-DMPC algorithm. Each cell in this table is a maximal robust positively invariant set, computed for a distinct parametrization of the input, state, and uncertainty constraint values. Thus, the invariant sets table is tailored specifically for a realistic vehicle platooning application, rather than the academic model of a cyber–physical multi-agent system from [18]. A vehicle platoon application is envisioned, consisting of four vehicles, dynamically coupled through the states. The model takes into account the dynamics of the vehicle, as opposed to the DMPC algorithms provided in [11,12,13], which were tested using a point-mass vehicle model. Each vehicle is locally controlled by an agent connected through an uni-directional channel, in a predecessor-following communication topology. The interaction between sub-systems is considered as state uncertainty for the nominal local model. This means that between consecutive vehicles, each agent in-charge needs to receive information from its predecessor and transmit data to its successor. Another key feature within the C-DMPC algorithm is the amount of information exchanged between agents, which is minimized by defining the uncertainties as boxes of fixed size, and transmitting only the bounding limits. Based on this uncertainty value, the feasibility of each local optimization problem is checked, and in case of infeasibility, a coalition between neighbouring agents is formed. The C-DMPC methodology is formulated to ensure zero-state minimization for the local and coalitional optimization problems, which is described in a more straightforward manner than in [18], where an integral state is introduced to ensure zero offset reference tracking. The robustness of the algorithm is analysed in various simulation tests, using a homogeneous platoon, with different initial conditions, both homogeneous and heterogeneous, under the same optimization parameters and constraints. The string stability of the platoon is analysed a posteriori, i.e., by verifying that the condition from [34] is satisfied on the obtained results, rather than imposing it as a constraint in the optimization problem. Moreover, the string stability of the platoon is investigated taking into account the nature of the proposed C-DMPC control framework. When a coalition is formed, the agents inside are treated as one entity with respect to the remaining agents outside of the coalition, which is different from the string stability analysis provided in a DMPC context in [35].

Taking all the above mentioned into considerations, the main contributions for this work, when compared to our previous papers and state-of-the-art literature are as follows:

1. A novel C-DMPC methodology, suitable for a state-coupled vehicle platooning application is developed.
2. The control performances of the algorithm were analysed for simulation tests using a realistic vehicle platoon model from the literature.
3. Both the robustness of the algorithm and the string stability of the platoon were investigated.

The remainder of this paper is structured as follows: Section 2 introduces the problem formulation, while the details regarding the computation of the invariant sets table are presented in Section 3. A summary of the C-DMPC method is provided in Section 4, followed by the simulation results and discussion given in Section 5. The conclusions and future work ideas are presented in Section 6.

*Notation:*N denotes the set of natural numbers; Z denotes the set of integer numbers; $Z_{+}$ denotes the set of positive integer numbers; R denotes the set of real numbers; X denotes a closed convex polytope in H-representation, written as a matrix inequality (X={x∈R:Ax≤b); ${∥.∥}_{1}$ denotes the 1-norm; and ${∥.∥}_{\infty }$ denotes the *∞*-norm.

## 2. Problem Formulation

Let us consider a multi-agent system consisting of *N* interconnected pairs ($S_{i}$, $A_{i}$), ∀i∈N, where N denotes the set {1,…,N}⊂N, $S_{i}$ denotes the sub-system and $A_{i}$ denotes the agent (or local controller) in charge of $S_{i}$. In the remaining of this paper, we consider that all sub-systems $S_{i}$ have a nominal model with state uncertainty, described as follows:

(1) $$\begin{array}{ccc} x_{i}\left(k+1\right) & = & A_{i,i}x_{i}\left(k\right)+B_{i,i}u_{i}\left(k\right)+w_{i}\left(k\right)\\ w_{i}\left(k\right) & = & A_{i,i-1}x_{i-1}\left(k\right) \end{array}$$

where $x_{i}\in R^{n_{i}}$ is the state, $u_{i}\in R^{m_{i}}$ is the input, $w_{i}\in R^{p_{i}}$ is the state uncertainty and $y_{i}\in R^{q_{i}}$ is the output. $A_{i,i}$, $B_{i,i}$, $A_{i,i-1}$ and $C_{i}$ are matrices with adequate dimensions. $n_{i}$, $m_{i}$, $p_{i}$ and $q_{i}$ are the number of states, inputs, state uncertainties and outputs, respectively, and $k\in Z_{+}$ is the discrete-time instant.

It is noteworthy to mention that in (1) the nominal model contains a state uncertainty $w_{i}$, defined based on the dynamical coupling of the sub-systems. Since all the sub-systems $S_{i}$, ∀i∈N, are unidirectionally interconnected, the relevant information is received from each predecessor denoted with index i−1.

Consider linear inequality constraints for the states, inputs and uncertainties defined with:

(2) $$x_{i}\in X_{i},\;u_{i}\in U_{i},\;w_{i}\in W_{i},\;\forall i\in N,$$

where $X_{i}$, $U_{i}$ and $W_{i}$ denote the polytopes obtained from the state, input and uncertainty constraints, respectively.

Further on, the local optimization problem solved by each agent $A_{i}$, ∀i∈N, is described in the min-max framework, by minimizing the control input for the maximum level of uncertainty, which will be received from its predecessor agent. Thus, the non-cooperative DMPC cost function solved at each sampling instant is as follows:

(3) $$\begin{array}{cc} J_{i}\left(x_{i}^{0}\right) & =\min_{\begin{array}{c} u_{i}^{0},\ldots ,u_{i}^{N_{p}-1}\\ {\hat{x}}_{i}^{\max} \end{array}}\max_{w_{i}^{0},\ldots ,w_{i}^{N_{p}-1}}\\ \; & ∥R_{w_{i}}{\hat{x}}_{i}^{\max}{∥}_{1}+\sum_{l=0}^{N_{p}-1}∥x_{i}^{l}{∥}_{1}+∥R_{u_{i}}u_{i}^{l}{∥}_{1}+{∥x_{i}^{N_{p}}∥}_{1}\\ s.\;t.\;(1)\\ \; & x_{i}^{\min}\leq x_{i}^{l}\leq {\hat{x}}_{i}^{\max}\leq x_{i}^{\max},\;l=1,\ldots ,N_{p}-1\\ \; & x_{i}^{N_{p}}\in \Omega_{i}\\ \; & u_{i}^{\min}\leq u_{i}^{l}\leq u_{i}^{\max},\;l=0,\ldots ,N_{p}-1\\ \; & w_{i}^{l}\leq w_{i}^{\max} \end{array}$$

with the following notations for sub-system $S_{i}$: $x_{i}^{l}=x_{i}\left(k+l|k\right)$ is the output predictor computed at sample time *k*, for time k+l, within the prediction horizon $N_{p}$, $l\in \{1,\ldots ,N_{p}\}$; the output trajectory is obtained recursively using model (1), the state measurement $x_{i}^{0}=x_{i}\left(k\right)$ at time *k*, the input trajectory $u_{i}^{l}=\left\{u_{i}^{0},\ldots ,u_{i}^{N_{p}-1}\right\}$ and the uncertainty trajectory $w_{i}^{l}=\left\{w_{i}^{0},\ldots ,w_{i}^{N_{p}-1}\right\}$, i.e., $W_{i}=A_{i,i-1}X_{i-1}$ received from agent $A_{i-1}$; $x_{i}^{N_{p}}$ is the value for the state predictor, for time $k+N_{p}$; $x_{i}^{\max}$, $u_{i}^{\max}$, $w_{i}^{\max}$ are the maximum limits for the state, input and the uncertainty sequences, respectively; $x_{i}^{\min}$, $u_{i}^{\min}$ are the minimum limits for the state and input sequences, respectively; $R_{u_{i}}\in R^{m_{i}\times m_{i}}$ and $R_{w_{i}}\in R^{n_{i}\times n_{i}}$ are the weight matrices for the input trajectory, and the optimization variable ${\hat{x}}^{\max}$ that acts as a self-imposed limit for the state sequence. After the local optimization problem is solved, the self-imposed value will be transmitted to the neighbour, which will use it as a boundary for the uncertainty variable. In this manner, each local agent is assured that the actual uncertainty trajectory (which is not communicated) is bounded at the received value.

The set $\Omega_{i}$ is a maximal robust positively invariant set for the output terminal constraint, and its computation will be detailed in the following.

## 3. Computation of the Maximal Robust Positively Invariant Set

In this section, the computation of the maximal robust positively invariant set $\Omega_{i}$, ∀i∈N, which will be used as a terminal output region $x_{i}^{N_{p}}\in \Omega_{i}$ is described. The procedure was initially presented in [19] and particularized in [18] for the sub-system model with an integral additional state.

Hereafter, a short summary is provided, followed by details regarding the computation of the invariant sets.

Let us consider the following assumptions:

- Each sub-system $S_{i}$, ∀i∈N, is modelled by (1) and subject to constraints (2);
- For the proposed coalitional framework, in the case of communication network failure, the information exchange between neighbouring agents stops. To ensure the feasibility of the control architecture, the invariant sets are tailored for the nominal model (in which the uncertainty $w_{i}$ is zero);
- A local linear feedback $u_{i}=K_{i}x_{i}$ that is computed to guarantee that the stability of the closed loop is a-priory determined. In this paper, the state feedback matrix $K_{i}$ is computed with two methods: (i) using Ackermann’s formula [36] and (ii) through the minimization of a linear-quadratic cost function [37].

For the sub-system (1) subject to the constraints (2), the set $\Omega_{i}$ is maximal robust positively invariant if [18,19,38]:

(4) $$\begin{array}{cc} & x_{i}\in \Omega_{i}\to \left(A_{i,i}+B_{i,i}K_{i}\right)x_{i}+w_{i}\in \Omega_{i},\\ & K_{i}x_{i}\in U_{i},\;\Omega_{i}\subseteq X_{i},\;\forall w_{i}\in W_{i} \end{array}$$

Hereafter, the pseudo-code algorithm provided in [18], which describes how to compute the predefined invariant set table is summarized and will be further detailed.

As previously mentioned, during the initialization phase, each agent $A_{i}$, ∀i∈N, calculates a table of invariant sets $\Omega_{i}$ by applying Algorithm 1.


**Algorithm 1**

**For**

$\alpha =u^{\max}:-step_{\alpha }:u^{\min}$


**For**

$\beta =w^{\min}:step_{\beta }:w^{\max}$

1. Compute the inequality constraints:
$$\begin{array}{c} A_{u}u\leq \alpha b_{u};\;A_{w}w\leq \beta b_{w};\;A_{x}x\leq b_{x} \end{array}$$
2. Compute the robust positively invariant set:
$$\begin{array}{c} \Omega (A,B,K,A_{u},b_{u},A_{w},b_{w},A_{x},b_{x}) \end{array}$$
3. Save the information α, β, Ω

**end**

**end**


Further on, several computational details regarding the second step from Algorithm 1 (i.e., how to compute the robust positively invariant set) are illustrated. For simplicity, the indices are omitted:

1. Compute the closed loop system matrix using the sub-systems model matrices *A*, *B*, and control feedback gain *K*;
2. Re-write the input inequality constraints from Algorithm 1, step 1, i.e., described in matrix form using matrix $A_{u}$ and vector $b_{u}$ with adequate dimensions, in the state variable *x* using u=Kx;
3. Aggregate state and input constraints from step 2 in a single polytope;
4. Compute the uncertainty polytope using the uncertainty constraints from Algorithm 1, step 1;
5. Iteratively, check the invariance order by executing the following:
  (a) Subtract the uncertainty polytope from the aggregated polytope from step 3;
  (b) Compute the polytope that, from the previous iteration, using the dynamics of the control loop, arrived in the current region (i.e., defined as step 5(a));
  (c) Intersect the two polytopes obtained at steps 5(a) and 5(b);
  (d) Compare the result of the polytopes intersection—from step 5(c) with the aggregated constraint polytope from step 3;
  (e) ***If*** the invariant set has changed
    *then:* Update the constraint polytope (with the value computed from step 5(c)) and continue the iterations;
    *else:* Save the invariant set and end the iterations.

It is worth mentioning that the maximal robust positively invariant set is computed recursively (as shown in step 5) for a finite number of iterations. If by the end of those, the desired region is not found, then the algorithm will return an empty set, meaning that for the particular combination of constraints values, there is no available invariant set (i.e., the corresponding cell in the table is empty).

## 4. Coalitional Distributed Model Predictive Control (C-DMPC) Algorithm

In this paper, a novel C-DMPC methodology was designed for sub-systems coupled through the states, which must exchange the state-uncertainty maximum value. Moreover, the algorithm which was firstly introduced in [18] for cyber–physical systems, was tailored in this work for a vehicle platooning application, and customized to ensure zero-state minimization. Hereafter, a concise summary is provided:

- At each sampling time, each agent solves a default robust min-max non-cooperative DMPC problem with state-uncertainty. If the local solution is infeasible, due to the uncertainty value received from its neighbour, then the coalition procedure between the agents is activated ad hoc without a supervisor level.
- Inside a coalition 𝒞, the local models of each involved sub-systems are merged, obtaining the following coalition model (written for simplicity without sub-script indices):
  (5) $$\begin{array}{ccc} x_{C}\left(k+1\right) & = & A_{C}x_{C}\left(k\right)+B_{C}u_{C}\left(k\right)+w_{C}\left(k\right)\\ w_{C}\left(k\right) & = & \sum_{j\in N_{C}}A_{C}^{j}x_{j}\left(k\right) \end{array}$$
  where $x_{C}$, $u_{C}$, $w_{C}$ are the aggregated state, input and uncertainty vectors of the coalition C (e.g., $x_{C}={\left[x_{i}\right]}_{i\in C}$). Moreover, the matrices $A_{C}$, $B_{C}$, $A_{C}^{j}$ and $C_{C}$ are computed according to the aggregation. The set $N_{C}$ contains the neighbouring agent for C neighbour, i.e., the predecessor sub-system for the sub-systems included in the coalition. For example, a coalition between agents $A_{3}$ and $A_{4}$ remains state-coupled with $A_{2}$, which is outside the coalition and sends information to $A_{3}$. It follows that, the constraint sets for a given coalition C are obtained as the union of the constraints sets (2) corresponding to each agent $A_{i}$, i∈C:
  (6) $$\begin{array}{c} x_{C}\in X_{C}=\underset{i\in C}{\prod }X_{i},\;\;u_{C}\in U_{C}=\underset{i\in C}{\prod }U_{i},\;\;w_{C}\in W_{C}=\underset{i\in C}{\prod }W_{i}.\;\; \end{array}$$
  where $X_{C}$, $U_{C}$ and $W_{C}$ are the state, input and uncertainty constraints polytopes for coalition C, respectively.
- A coalition once formed is active only for a single sampling period and can gradually increase its size. Hence, the feasibility for the coalition optimization problem is verified. If the solution is infeasible, this means that the received uncertainty value is too large, and an agent from the neighbourhood must be incorporated in the coalition, to decrease the uncertainty value. For example, if the coalition between $A_{3}$ and $A_{4}$ has an infeasible solution, then, in the same sampling time, agent $A_{2}$ is included in the existing coalition. Afterwards, the optimal solutions are re-calculated, for both the coalition and the agents outside the coalition, and the feasibility is re-evaluated. Following this reasoning, in the extreme case, all the agents from the network can be gradually included in a single coalition. This represents the particular case of a centralized optimization problem. Inside a coalition C, the following optimization problem is minimized:
  (7) $$\begin{array}{ccc} J_{C}\left(x_{C}^{0}\right) & = & \min_{\begin{array}{c} u_{C}^{0},\ldots ,u_{C}^{N_{p}-1}\\ {\hat{x}}_{C}^{\max} \end{array}}\max_{w_{C}^{0},\ldots ,w_{C}^{N_{p}-1}}\\ & & ∥R_{w_{C}}{\hat{x}}_{C}^{\max}{∥}_{1}+\sum_{l=0}^{N_{p}-1}∥x_{C}^{l}{∥}_{1}+∥R_{u_{C}}u_{C}^{l}{∥}_{1}+{∥x_{C}^{N_{p}}∥}_{1}\\ s.\;t.(5)\\ x_{C}^{\min} & \leq & x_{C}^{l}\leq {\hat{x}}_{C}^{\max}\leq x_{C}^{\max},\;l=1,\ldots ,N_{p}-1\\ x_{C}^{N_{p}} & \in & \Omega_{C}\\ u_{C}^{\min} & \leq & u_{C}^{l}\leq u_{C}^{\max},\;l=0,\ldots ,N_{p}-1\\ w_{C}^{l} & \leq & w_{C}^{\max} \end{array}$$
  where, the weight matrices $R_{u_{C}}$ and $R_{w_{C}}$ are block diagonal; $\Omega_{C}$ is the aggregated terminal set; $x_{C}^{0}$ is the aggregated vector containing the corresponding initial state values; $x_{C}^{\max}$, $u_{C}^{\max}$, $w_{C}^{\max}$ are the aggregated maximum limits for the state, input and the uncertainty sequences, respectively; $x_{C}^{\min}$, $u_{C}^{\min}$ are the aggregated minimum limits for the state and input sequences, respectively; $u_{C}^{l}=\left\{u_{C}^{0},\ldots ,u_{C}^{N_{p}-1}\right\}$, $w_{C}^{l}=\left\{w_{C}^{0},\ldots ,w_{C}^{N_{p}-1}\right\}$ and $x_{C}^{l}$ are the aggregated input, uncertainty and state trajectories of the coalition C; the optimization variable ${\hat{x}}_{C}^{\max}$ is the aggregated self-imposed limit for the aggregated state sequence.
- Between neighbouring agents, only the self-imposed optimization variable ${\hat{x}}^{\max}$ from (3) is shared. In this manner, although the sub-systems are state-coupled, the coupling signals are considered as local uncertainties, and a robust optimization problem is defined using the shared information as the uncertainty limit. With this value received from the neighbour, each local agent searches for a robust positively invariant set in the predefined table, computed in the initialization phase of the algorithm (see Section 2). If there is no pre-computed terminal region $\Omega_{i}$ which can include the received uncertainty, then the local optimization problem becomes infeasible and the coalition procedure is started.

In what follows, a summarized pseudo-code for the coalitional DMPC, which uses the invariant sets as terminal regions is provided. Starting from the algorithm provided in [18], the following novel algorithm is obtained for the current system configuration, namely state-coupled sub-systems, which at each iteration must exchange the state uncertainty polytope.

**Remark 1.**  *In Algorithm 2, step 6, the local feasibility is verified for all agents in the network. When more than one agent has an infeasible local solution, a sub-unitary random coalition priority is given to each one of them. This means that, to avoid conflicts and subjectivity, all agents in need of a coalition, i.e., due to infeasibility, have an equal chance of initially entering in a coalition. However, after this random coalition initialization start-up, the methodology sequentially increases the size of the coalition when needed, always starting from coalitions of 2 agents.*

**Algorithm 2**
Initialization: For each agent $A_{i}$, ∀i∈N, compute a table $T_{i}$, with potential terminal sets $\Omega_{i}$.At each sampling time *k*, each agent $A_{i}$, ∀i∈N, receives the local state value and performs the following steps:1. Computes the uncertainty polytope using default limit values for the state-uncertainty constraints:
$$\begin{array}{ccc} W_{i} & = & A_{i,i-1}X_{i-1}^{0}\\ X_{i-1} & = & [x_{i-1}^{\max,0};-x_{i-1}^{\max,0}]. \end{array}$$
2. Searches in the predefined table $T_{i}$ for a terminal set $\Omega_{i}^{0}$ that includes the default uncertainty $W_{i}\subseteq \Omega_{i}^{0}$.
3. Solves the local optimization problem (3) and obtains the optimal values $U_{i}^{*,0}$, ${\hat{x}}_{i}^{\max,0}$ using the default values $\Omega_{i}=\Omega_{i}^{0}$
             for the terminal set and the uncertainty constraint limit ($w_{i}^{\max}=x_{i-1}^{\max,0}$). 4. Broadcasts to its successor the local optimization value ${\hat{x}}_{i}^{\max,0}$ and receives the corresponding value ${\hat{x}}_{i-1}^{\max,0}$ from its             predecessor.
5. Repeats Steps 1–3 using the uncertainty constraint value received in Step 4.
6. Checks the feasibility of the local optimization problem:
***If*** the optimization problem from Step 5 is feasible:
*then*: Coalitions between agents are not necessary. Each local agent $A_{i}$ sends to its sub-system $S_{i}$, the first value from the             optimal trajectory $U_{i}^{*}$;
*else*: Coalitions between agents are necessary. The coalition model is defined as in (5), by aggregating the models of           the sub-systems involved in the coalition. Moreover, the optimization problem (3) is redefined in terms of the           coalition and is solved.
              ***If*** all the optimization problems (inside and outside the coalition) are feasible:
              *then*: The existing coalition was successful and can be dissolved after every sub-system $S_{i}$ receives the first                         value from the optimal trajectory $U_{i}^{*}$;
              *else*: The existing coalition was not successful. Another agent must be included in the existing coalition.
7. End algorithm.

**Remark 2**  ([18,19]). *Within Algorithm 2, the feasibility of the coalitional control problem is ensured because $W_{C}\subseteq \prod_{i\in C}W_{i}$, $U_{C}=\prod_{i\in C}U_{i}$ and $\Omega_{C}=\prod_{i\in C}\Omega_{i}$. Keeping the coalition stable is guaranteed by the terminal constraint set, calculated as the Minkowski sum of the polytopes for each individual agent of the coalition. As long as initially all of the local optimization problems can be solved in a decentralized fashion, the algorithm is recursively feasible.*

## 5. Simulation Results

In this section, the C-DMPC methodology is tested in simulation for a vehicle platooning application described in Section 5.1 and the obtained results are provided in Section 5.2. Moreover, the string stability analysis of the platoon is provided in Section 5.3.

### 5.1. Platooning Application Setup

**Figure 1:**
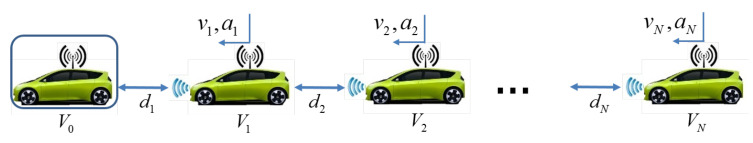
Schematic diagram of a vehicle platoon.

In this subsection, the C-DMPC methodology is tested in simulation for a N=4 vehicle platooning application, as described in [39]. Each vehicle from the platoon is modelled with the following continuous-time dynamical model [40]:

(8) $$\begin{array}{c} \underset{{\dot{x}}_{i}}{\underbrace{\left[\begin{array}{c} \Delta {\dot{d}}_{i}\\ \Delta {\dot{v}}_{i}\\ {\dot{a}}_{i} \end{array}\right]}}=\underset{A_{i,i}}{\underbrace{\left[\begin{array}{ccc} 0 & 1 & -h\\ 0 & 0 & 1\\ 0 & 0 & -1/\tau_{i} \end{array}\right]}}\underset{x_{i}}{\underbrace{\left[\begin{array}{c} \Delta d_{i}\\ \Delta v_{i}\\ a_{i} \end{array}\right]}}+\underset{B_{i,i}}{\underbrace{\left[\begin{array}{c} 0\\ 0\\ 1/\tau_{i} \end{array}\right]}}u_{i}+\underset{A_{i,i-1}}{\underbrace{\left[\begin{array}{ccc} 0 & 0 & 0\\ 0 & 0 & 1\\ 0 & 0 & 0 \end{array}\right]}}x_{i-1} \end{array}$$

where $\Delta d_{i}\left(t\right)=d_{i}\left(t\right)-d_{r,i}\left(t\right)$ is the distance between platoon vehicles, and $d_{r,i}\left(t\right)=r-hv_{i}\left(t\right)$ is the desired relative distance, computed taking into account the standstill distance *r* and the time headway value *h*. The relative velocity between two consecutive vehicles is $\Delta v_{i}\left(t\right)=v_{i-1}\left(t\right)-v_{i}\left(t\right)$, and the relative acceleration is $\Delta {\dot{v}}_{i}\left(t\right)=a_{i-1}\left(t\right)-a_{i}\left(t\right)$. Note that the vehicle’s model (8) is coupled through the states. The time constant of the dynamics for the vehicle’s engine is $\tau_{i}$, while $u_{i}$ is its input, defined as the desired acceleration for the vehicle. In Figure 1, a schematic diagram for a vehicle platooning application is provided, in which, the leader denoted $V_{0}$ travels with a constant velocity and has $a_{0}=0$ and $u_{0}=0$, and is considered a virtual leader which is not optimized. Thus, in this paper, we control only the follower vehicles from the platoon, denoted with $V_{i}$, ∀i∈{1,…,4}.

For simulation purposes, all followers are considered identical, with $\tau_{i}=0.1$, ∀i∈{1,…,4}, and the headway time value h=1.5. The vehicle’s model (8) was discretized with the sampling period Ts=0.1 s.

The optimization parameters used in the cost function (3) are: the prediction horizon $N_{p}=26$ samples; the input and the uncertainty weights $R_{u_{i}}=0.1$ and $R_{w_{i}}=1$, respectively, ∀i∈{1,…,4}. These values were empirically selected to achieve the best results in the C-DMPC algorithm. This means that, the feasibility of the algorithm must be ensured at each sampling time, for one of the possible communication topologies.

The numerical values for the limit constraints described in (2) are as follows:

$$\begin{array}{cccc} \left\{\begin{array}{c} u_{i}^{\min}=-5\\ u_{i}^{\max}=5 \end{array}\right. & \left\{\begin{array}{c} y_{i}^{\min}=-7\\ y_{i}^{\max}=7 \end{array}\right. & \left\{\begin{array}{c} w_{i}^{\max}=1\\ w_{i}^{\min}=-1 \end{array}\right. & \forall i\;\in \;\{1,\ldots ,4\}. \end{array}$$

Please note that, in order to compute the table with invariant sets, these values were taken into account.

To compute the feedback control laws $K_{i}$, ∀i∈{1,…,4}, using the vehicle model (8), two case-studies were analysed, corresponding to the two methods that were used, namely:

- Case 1: classical state-feedback control, based on the Ackermann’s formula [36], obtaining:
  (9) $$\begin{array}{c} K=K_{1}=K_{2}=K_{3}=K_{4}=\left[0.5374\;0.0263\;0.3628\right]. \end{array}$$
  Please note that, this feedback gain matrix was obtained imposing identical desired closed-loop transient performance for each sub-system, namely an overshoot value of 5% and settling time of five time units.
- Case 2: minimization of a linear-quadratic cost function, by solving a discrete-time Riccatti Equation [37]:
  (10) $$\begin{array}{c} \tilde{K}={\tilde{K}}_{1}={\tilde{K}}_{2}={\tilde{K}}_{3}={\tilde{K}}_{4}=\left[0.4044\;0.6045\;-0.1562\right]. \end{array}$$
  It is worthy to mention that, these matrices were computed using the same weight matrix as in the solved optimization problem, i.e., $R_{u_{i}}=5$.

**Figure 2:**
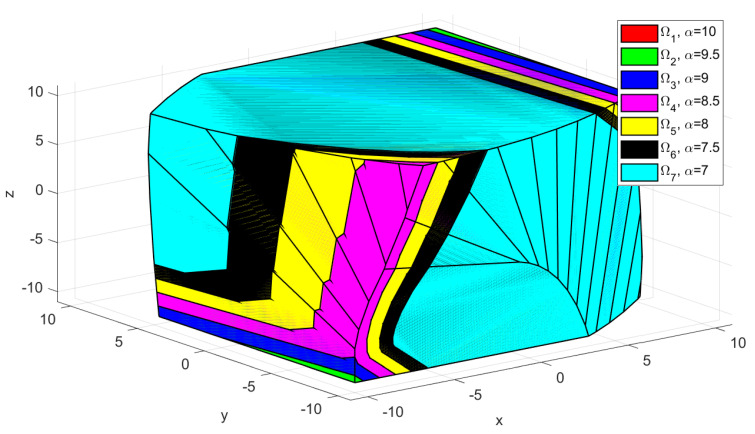
Case 1—Depiction of the predefined invariant sets corresponding to sub-system S1, computed for uncertainty constraint limit value β=0.1.

**Figure 3:**
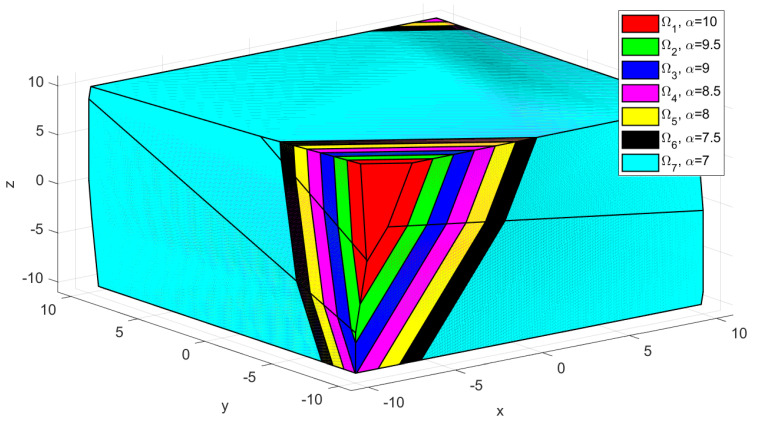
Case 2—Depiction of the predefined invariant sets corresponding to sub-system S1, computed for uncertainty constraint limit value β=0.1.

The invariant sets obtained for sub-system $S_{1}$ using Algorithm 1, from Section 3 are depicted in Figure 2 and Figure 3. Those sets were computed using the following numerical values: $u^{\max}=10$, $w^{\max}=10$, $w^{\min}=0.1$, $step_{\alpha }=step_{\beta }=0.5$. The polytopes computed using the feedback matrix *K* from Case 1 are denoted with $\Omega_{i}$, and plotted in Figure 2. Similarly, the polytopes calculated using the feedback matrix $\tilde{K}$ from Case 2 are denoted with ${\tilde{\Omega }}_{i}$ and depicted in Figure 3. In both figures, the largest invariant set (i.e., corresponding to the maximum input constraint value $\alpha =u^{\max}=10$ and the minimum uncertainty β=0.1) is depicted in red colour. As expected, the larger possible invariant set that can be used in the terminal constraint definition includes the other smaller invariant sets. This is especially visible in Figure 3, in which the smaller polytopes, obtained when the input constraint is decreased are gradually “cropped” from the dimension of the red polytope. Both Figure 2 and Figure 3 provide a graphical representation for the invariant set table computed in the initialization phase of the algorithm, i.e., for each cell in the table, a coloured polytope is depicted. According to the design of Algorithm 1 from Section 3, several invariant sets are computed, for different parametrizations of the input and uncertainty constraints limits, via α and β values. Thus, the larger invariant set polytope (i.e., corresponding to the maximum input constraint value $\alpha =u^{\max}=10$ and the minimum uncertainty β=0.1) is denoted with $\Omega_{1}$ (see Figure 2 and Figure 3). However, as the input limit decreases, also the invariant sets have smaller dimensions, which are included in the larger set. Note that, the computed invariant sets are three dimensional, because the state variable for the vehicle model (8) has three values, and can be plotted as convex hull (ref. Figure 2 and Figure 3).

**Remark 3.**  *Algorithm 1, from Section 3 was implemented in MATLAB R2020b using the YALMIP toolbox [41]. For each input, state and uncertainty constraint set values, a maximum of 100 iterations were computed to search for the robust positively invariant set.*

**Remark 4.**  *The computational complexity of Algorithm 1, is related to the number of iterations required to compute each robust positively invariant set $\Omega_{i}$ from table $T_{i}$. This value gives the invariance order for each $\Omega_{i}$. Although this is computed only once and outside of the procedure which solves the optimization (see Initialization from Algorithm 2), the necessary computational time is non-trivial. This is influenced by the used parametrization, which should be chosen according to the sub-system’s constraint limits (i.e., the values α and β, and their corresponding step size values $step_{\alpha }=step_{\beta }$). Furthermore, the feedback gain matrix K has a significant impact on the algorithm complexity, which can be seen in the invariance orders for each $\Omega_{i}$ set. Hence, under the same parametrization, using the feedback matrix K from Case 1, 16 invariant sets were obtained, with invariance orders ranging from 14 to 45 iterations. Furthermore, using the feedback matrix $\tilde{K}$ from Case 2, 17 invariant sets were obtained, with invariance orders ranging from 3 to 36 iterations. Note that, for simplicity, only the first 7 polytopes, in the descending order with respect to the α value, denoted with $\Omega_{i}$, ∀i∈{1,…,7}, are plotted.*

### 5.2. Illustrative Results

In the local optimization problem formulated in (3), the control goal was to regulate all local states to zero. From a vehicle platooning perspective, in which each vehicle is modelled with (8), this results in a vehicle platoon which travels with a constant speed and implicitly with 0 acceleration, and maintains a constant desired inter-vehicle distance. A multitude of tests were performed on the homogeneous platoon defined in Section 5.1. These were conducted using both homogeneous and heterogeneous initial conditions $x_{i}^{0}$, for each vehicle ∀i∈{1,…,4}, with numerical values selected within the imposed constraint limits. However, for brevity, the most representative tests are provided, with the following initial values:

**Remark 5.**  *Two simulations were performed for each test, using the invariant set tables $T_{i}$, computed with the feedback matrix K from (9) and $\tilde{K}$ from (10), respectively. The simulation results obtained using the invariant sets $\Omega_{i}$ computed with the feedback matrix $\tilde{K}$ from Case 2, were almost identical to the ones computed with the feedback matrix K from Case 1, and for brevity were not included in the paper.*

The performance of the proposed C-DMPC algorithm was studied for all the simulation tests mentioned in Table 1, and is given in Table 2. This evaluation was performed computing the cumulative cost index in norm-1, using the state vectors of each vehicle $V_{i}$, ∀i∈{1,…,4}, from the platoon, i.e., $J_{V_{i}}=∥\Delta d_{i}{∥}_{1}+∥\Delta v_{i}{∥}_{1}+{∥a_{i}∥}_{1}$. Moreover, for an overall view, the cumulative cost index for each simulated test ${\mathrm{Test}}_{j}$, ∀j∈{1,…,8}, is also computed, i.e., $J_{{\mathrm{Test}}_{j}}=\sum_{i=1}^{4}J_{V_{i}}$. The computational time for each simulation test is also provided. The consistence of the performance indices values effectively illustrate the robustness of the proposed C-DMPC algorithm.

**Remark 6.**  *The proposed algorithm was tested in simulation and was designed to ensure satisfactory control performances for a vehicle platooning application. The simulations were performed using MATLAB R2020b on Windows 10, 64-bit Operating System with a laptop Intel Core i7-9850H CPU @ 2.60 GHz460 and 16 GB RAM. Thus, the algorithm was not yet optimized to be executed on embedded devices, and to be tested in a real-time setup, but this is a subject of future work.*

Hereafter, for brevity, only the simulation results for two tests from Table 1 are presented, namely starting from: (i) the homogeneous initial values from ${\mathrm{Test}}_{4}$, and (ii) the heterogeneous initial values from ${\mathrm{Test}}_{8}$.

**Figure 4:**
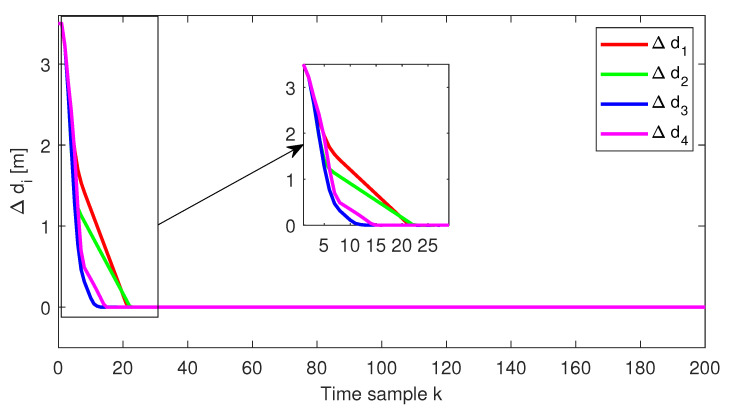
${\mathrm{Test}}_{4}$—Distance error for each platoon vehicle.

**Figure 5:**
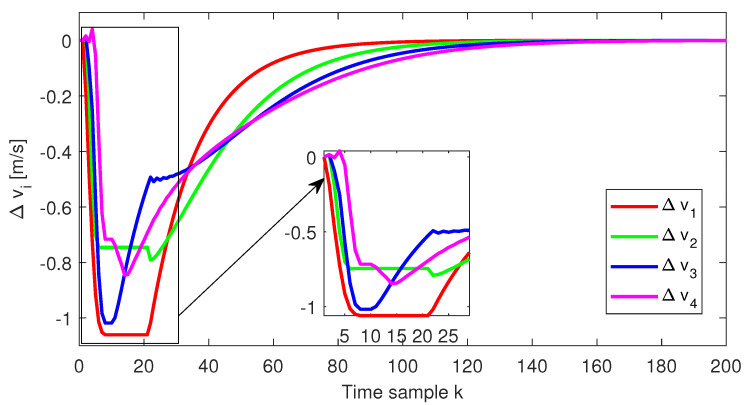
${\mathrm{Test}}_{4}$—Relative speed for each platoon vehicle.

**Figure 6:**
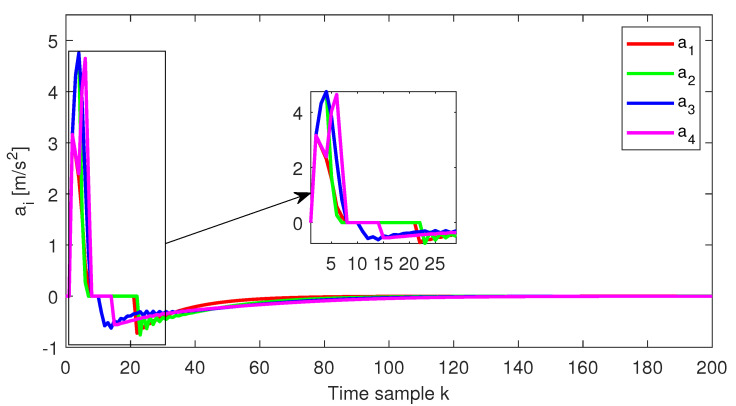
${\mathrm{Test}}_{4}$—Acceleration for each platoon vehicle.

**Figure 7:**
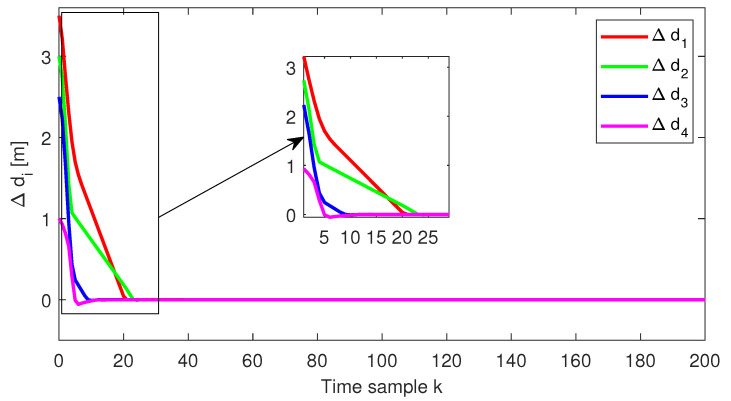
${\mathrm{Test}}_{8}$—Distance error for each platoon vehicle.

**Figure 8:**
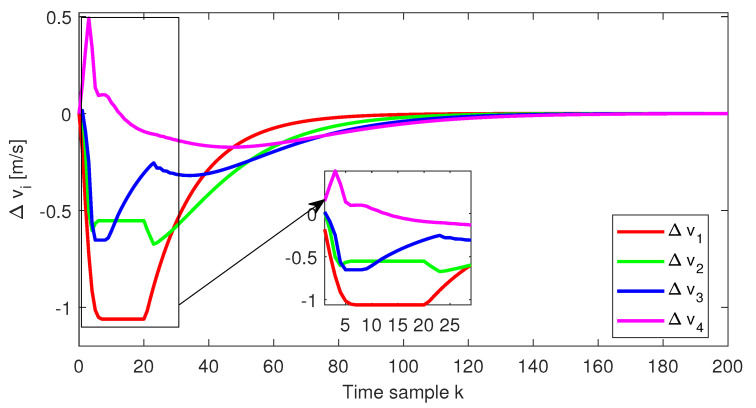
${\mathrm{Test}}_{8}$—Relative speed for each platoon vehicle.

**Figure 9:**
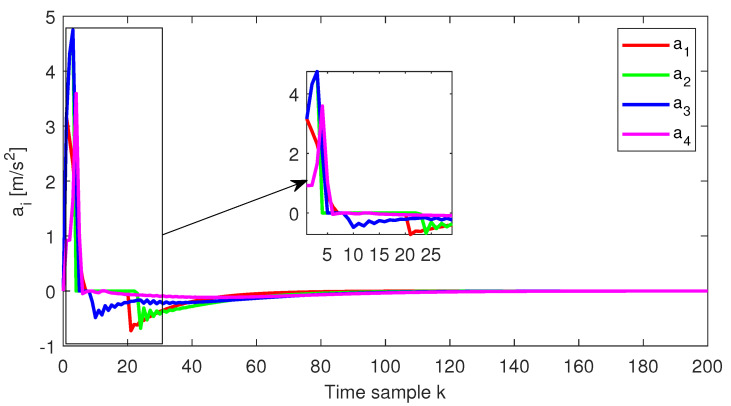
${\mathrm{Test}}_{8}$—Acceleration for each platoon vehicle.

The relative distance error, the relative speed and the acceleration for each follower vehicle, respectively, obtained for the initial conditions from ${\mathrm{Test}}_{4}$ (ref. Table 1), are depicted in Figure 4, Figure 5 and Figure 6. Furthermore, the same variables are depicted in Figure 7, Figure 8 and Figure 9, obtained for the initial conditions from ${\mathrm{Test}}_{8}$ (ref. Table 1).

**Figure 10:**
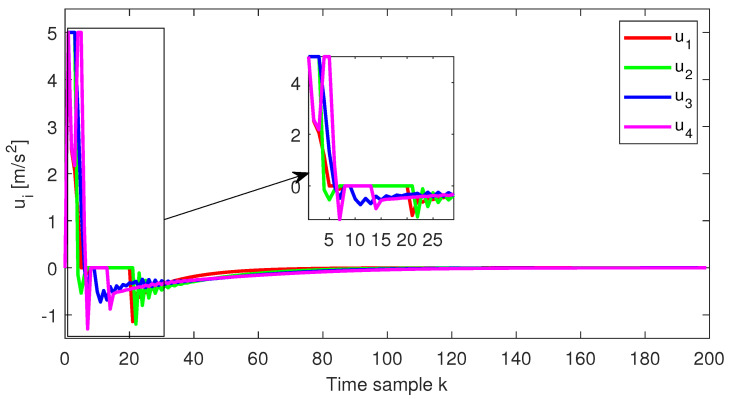
${\mathrm{Test}}_{4}$—Control input for each platoon vehicle.

**Figure 11:**
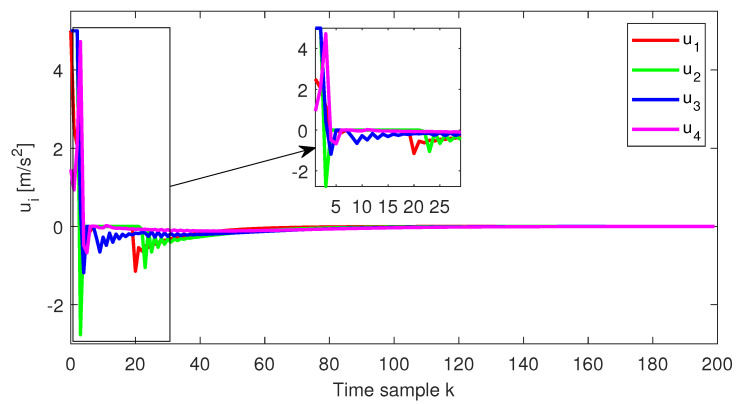
${\mathrm{Test}}_{8}$—Control input for each platoon vehicle.

As expected, at the end of the simulation scenario, all the states and implicitly the inputs (see Figure 10 and Figure 11, respectively) reach the desired zero value.

From the C-DMPC point of view, a platoon consisting of 4 vehicles uni-directionally coupled in a leader-follower communication architecture, has eight possible communication frameworks, including coalitions of 2, 3 or 4 agents defined in Table 3 [18].

As previously mentioned, in the proposed C-DMPC algorithm, all agents first solve a robust min-max non-cooperative DMPC problem with state uncertainty, starting from given initial conditions. This can be seen as $C_{\emptyset }$ active, in which all agents are outside a coalition. If one or more local solutions are infeasible, then the coalition procedure starts and coalitions between different agents are formed.

**Figure 12:**
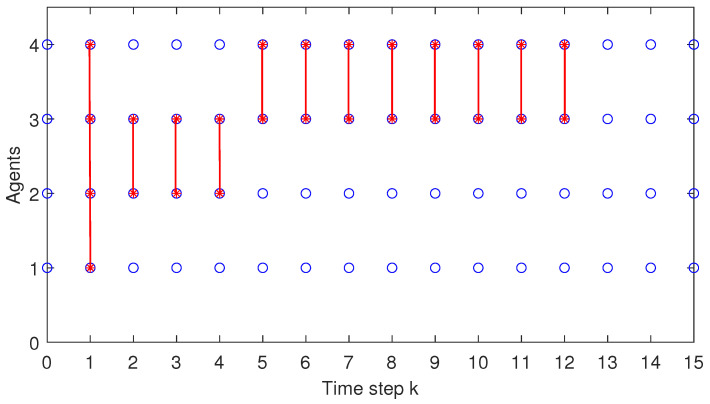
${\mathrm{Test}}_{4}$—Coalitions formed at each time instant.

**Figure 13:**
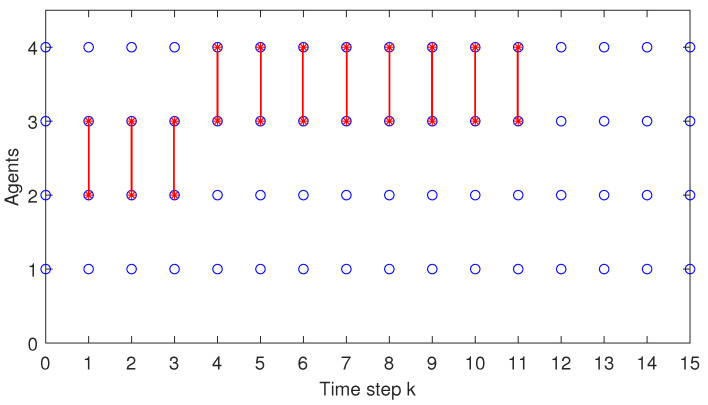
${\mathrm{Test}}_{8}$—Coalitions formed at each time instant.

When analysing both Figure 12 and Figure 13, one can remark that only in the beginning of the simulation test, i.e., for the first 12 samples (see Figure 12) and, respectively, 11 samples (see Figure 13), the coalitions are activated.

This is due to the fact that for the particular initial condition from the simulation test chosen for the platoon, some of the local problems are infeasible. Our proposed C-DMPC algorithm is designed such that, at each sampling time, when a local problem is infeasible, i.e., the min-max DMPC algorithm fails to reach a solution, a coalition is activated. Moreover, the algorithm always starts with the smallest coalition size, i.e., including two agents, and when needed, increases its dimension by sequentially including other neighbours, until the largest possible size is reached, i.e., see the activation of $C_{1234}$, at time step *k* = 1 in Figure 12. After the feasible solution is reached, the current coalition deactivates, and the procedure is repeated at the next sampling time. This is the reason that in Figure 12, at time step k=2, a smaller size coalition $C_{23}$ between agents $A_{2}$ and $A_{3}$ is activated. Coincidentally, in Figure 12, coalition $C_{23}$ is consecutively activated at time steps k=2,3 and 4, whereas coalition $C_{34}$ between $A_{3}$ and $A_{4}$ is activated from time samples k=5 to k=12. Afterwards, since both the acceleration state and the control input almost reach the desired zero value (see the detailed plot from Figure 6 and Figure 10), there is no need for the coalition activation. In this case, the uncertainty received from the predecessor vehicle is included in one of the predefined terminal sets, and the local problem is feasible. Hence, starting from time instant k=13 until the end of the simulation, the control architecture for all the platoon vehicles is min-max non-cooperative DMPC (or $C_{\emptyset }$). For a better visualisation, only the first 15 time samples are plotted in Figure 12. An agent inside a coalition is depicted with a red star marker within a blue circle, whereas for one outside a coalition, the corresponding blue circle is empty. When analysing Figure 13, the same reasoning is used and one can remark that coalition $C_{23}$ is activated from time samples k=1 to k=3, while coalition $C_{34}$ is activated from time samples k=4 to k=11. After that time-instant, no more coalitions are activated.

### 5.3. String Stability Analysis

The string stability of the vehicle platoon, which ensures that the inter-vehicle distance error does not amplify towards the end of the platoon is analysed using the formulation given in [34]:

(11) $${∥\Delta d_{i}∥}_{\infty }\leq \epsilon_{i}{∥\Delta d_{i-1}∥}_{\infty }$$

where $\Delta d_{i}$ and $\Delta d_{i-1}$ are the distance errors for two consecutive vehicles from the platoon, and $\epsilon_{i}\in \left(0,1\right)$ is a parameter.

To verify that the platoon is string stable, the property (11) was used a posteriori, similarly with the work in [35], by analysing the corresponding $\Delta d_{i}$ values obtained for each vehicle from the platoon. This was performed by computing the ϵ values for every two consecutive vehicles in the platoon, which according to (11), must be smaller or equal to 1, to ensure that the errors do not propagate towards the end of the platoon. Since the simulated platoon does not have a leader vehicle, the first investigation was performed between vehicles $V_{2}$ and $V_{3}$ by firstly computing $\epsilon_{3}$. Next, $\epsilon_{4}$ was computed between vehicles $V_{3}$ and $V_{4}$.

All the simulation tests starting from the initial conditions provided in Table 1 were investigated, and the results are given in Table 4.

However, one must take into account that our results are obtained in the C-DMPC framework. This means that when a coalition is activated, the vehicles inside the coalition must be viewed as a single entity, since the models are aggregated in the coalitional optimization problem. Thus, according to the coalitions activated in ${\mathrm{Test}}_{8}$ simulation, when $C_{23}$ is active, the distance error for $A_{3}$ is not taken into account, thus analysing only the states for vehicles 1, 2 and 4. Moreover, when $C_{34}$ activates, only the states for vehicles 1, 2 and 3 are meaningful, since $A_{4}$ is inside the coalition, and ‘coupled’ with $A_{3}$.

Hence, the string stability property for the proposed C-DMPC suitable for a platooning application is satisfied in all the investigated tests.

**Remark 7.**  *When analysing the string stability of a platoon controlled with a coalitional methodology, one must take into account the coalitions which are formed between the vehicles, which implicitly guarantee the feasibility of all the optimization problems.*

## 6. Conclusions

In this paper, a coalitional distributed model predictive (C-DMPC) methodology, suitable for a vehicle platooning application was proposed. Details regarding the computation of the maximal robust positively invariant sets, which were used as terminal regions in the C-DMPC algorithm were also provided. The control strategy was tested for a vehicle platooning application, consisting of a virtual leader and four follower vehicles, with a leader-follower uni-directional communication topology. The simulation results show the efficiency of the proposed coalitional DMPC algorithm, in which the coalitions between different agents are formed when needed (i.e., the local feasibility of the solution is lost). Otherwise, the control topology between vehicles is robust min-max non-cooperative DMPC with state uncertainties.

Future work will test the proposed C-DMPC algorithm in an experimental setup using a platoon of mobile robots.

## 7. Materials and Methods

The simulations from this work were performed using MATLAB R2020b on Windows 10, 64-bit Operating System on a laptop (Intel Core i7-9850H CPU @ 2.60 GHz and 16 GB RAM). The optimizations were implemented using the YALMIP toolbox [41].

## Figures and Tables

**Table 1:** Initial states values.

Test	$x_{1}^{0}$	$x_{2}^{0}$	$x_{4}^{0}$	$x_{4}^{0}$
${\mathrm{Test}}_{1}$	${\left[1.5\;0\;0\right]}^{T}$	${\left[1.5\;0\;0\right]}^{T}$	${\left[1.5\;0\;0\right]}^{T}$	${\left[1.5\;0\;0\right]}^{T}$
${\mathrm{Test}}_{2}$	${\left[2\;0\;0\right]}^{T}$	${\left[2\;0\;0\right]}^{T}$	${\left[2\;0\;0\right]}^{T}$	${\left[2\;0\;0\right]}^{T}$
${\mathrm{Test}}_{3}$	${\left[2.5\;0\;0\right]}^{T}$	${\left[2.5\;0\;0\right]}^{T}$	${\left[2.5\;0\;0\right]}^{T}$	${\left[2.5\;0\;0\right]}^{T}$
${\mathrm{Test}}_{4}$	${\left[3.5\;0\;0\right]}^{T}$	${\left[3.5\;0\;0\right]}^{T}$	${\left[3.5\;0\;0\right]}^{T}$	${\left[3.5\;0\;0\right]}^{T}$
${\mathrm{Test}}_{5}$	${\left[2\;0\;0\right]}^{T}$	${\left[1.5\;0\;0\right]}^{T}$	${\left[1\;0\;0\right]}^{T}$	${\left[0.5\;0\;0\right]}^{T}$
${\mathrm{Test}}_{6}$	${\left[2\;0\;0\right]}^{T}$	${\left[2\;0\;0\right]}^{T}$	${\left[1\;0\;0\right]}^{T}$	${\left[1\;0\;0\right]}^{T}$
${\mathrm{Test}}_{7}$	${\left[3\;0\;0\right]}^{T}$	${\left[2.5\;0\;0\right]}^{T}$	${\left[2\;0\;0\right]}^{T}$	${\left[1.5\;0\;0\right]}^{T}$
${\mathrm{Test}}_{8}$	${\left[3.5\;0\;0\right]}^{T}$	${\left[3\;0\;0\right]}^{T}$	${\left[2.5\;0\;0\right]}^{T}$	${\left[2\;0\;0\right]}^{T}$

**Table 2:** Performance indices. All the numerical values are multiplied with 1.0 × 10${}^{3}$.

Test	$J_{V_{1}}$	$J_{V_{2}}$	$J_{V_{3}}$	$J_{V_{4}}$	$J_{{\mathrm{Test}}_{j}}$	Sim Time (s)
${\mathrm{Test}}_{1}$	0.0357	0.0371	0.0354	0.0367	0.1449	2.7925
${\mathrm{Test}}_{2}$	0.0476	0.0496	0.0484	0.0493	0.1950	2.7627
${\mathrm{Test}}_{3}$	0.0620	0.0635	0.0635	0.0671	0.2561	3.1780
${\mathrm{Test}}_{4}$	0.0869	0.0900	0.0914	0.0948	0.3631	3.2602
${\mathrm{Test}}_{5}$	0.0476	0.0375	0.0241	0.0132	0.1224	3.3953
${\mathrm{Test}}_{6}$	0.0476	0.0496	0.0249	0.0245	0.1467	2.8173
${\mathrm{Test}}_{7}$	0.0745	0.0623	0.0528	0.0444	0.2340	3.0752
${\mathrm{Test}}_{8}$	0.0834	0.0747	0.0620	0.0340	0.2540	3.1732

**Table 3:** Possible communication topologies.

Coalition	Agent 1	Agent 2	Agent 3	Agent 4
$C_{\emptyset }$	-	-	-	-
$C_{12}$	$A_{1}$	$A_{2}$	-	-
$C_{123}$	$A_{1}$	$A_{2}$	$A_{3}$	-
$C_{23}$	-	$A_{2}$	$A_{3}$	-
$C_{234}$	-	$A_{2}$	$A_{3}$	$A_{4}$
$C_{1234}$	$A_{1}$	$A_{2}$	$A_{3}$	$A_{4}$

**Table 4:** String stability property.

Test	$\epsilon_{3}$	$\epsilon_{4}$
${\mathrm{Test}}_{1}$	1.0000	1.0000
${\mathrm{Test}}_{2}$	1.0000	1.0000
${\mathrm{Test}}_{3}$	1.0000	1.0000
${\mathrm{Test}}_{4}$	1.0000	1.0000
${\mathrm{Test}}_{5}$	0.6667	0.5000
${\mathrm{Test}}_{6}$	0.5000	1.0000
${\mathrm{Test}}_{7}$	0.8000	0.7500
${\mathrm{Test}}_{8}$	0.8333	0.4000

## Data Availability

Not applicable.

## Abbreviationbbreviations

The following abbreviations are used in this manuscript:

MPC	Model Predictive Control
DMPC	Distributed Model Predictive Control
C-DMPC	Coalitional Distributed Model Predictive Control
